# Retinal Vascular Assessment in Psoriasis: A Multicenter Study

**DOI:** 10.3389/fnins.2021.629401

**Published:** 2021-01-25

**Authors:** Niccolò Castellino, Antonio Longo, Matteo Fallico, Andrea Russo, Vincenza Bonfiglio, Gilda Cennamo, Federica Fossataro, Gabriella Fabbrocini, Anna Balato, Guglielmo Parisi, Rosa Maria D’urso, Francesco Lacarrubba, Maria Letizia Musumeci, Pietro Alosi, Francesco Petrillo, Giuseppe Micali, Teresio Avitabile, Michele Reibaldi

**Affiliations:** ^1^Department of Ophthalmology, University of Catania, Catania, Italy; ^2^Department of Experimental Biomedicine and Clinical Neuroscience, Ophthalmology Section, University of Palermo, Palermo, Italy; ^3^Public Health Department, University of Naples Federico II, Naples, Italy; ^4^Department of Neuroscience, Reproductive Sciences and Dentistry, University of Naples Federico II, Naples, Italy; ^5^Department of Advanced Biomedical Sciences, University of Naples Federico II, Naples, Italy; ^6^Department of Surgical Science, Dermatology Clinic, University of Catania, Catania, Italy; ^7^Department of Surgical Science, Eye Clinic, University of Turin, Turin, Italy

**Keywords:** psoriasis, vascular changes, retina, optical coherence tomography angiography, macula

## Abstract

**Purpose:**

To investigate the vascular status of the macula in psoriasis patients without history of ocular inflammation by Optical Coherence Tomography Angiography (OCTA).

**Methods:**

This prospective cross-sectional multicenter study included 55 psoriasis patients and 55 control healthy subjects. A complete eye examination and 6 mm × 6 mm OCTA imaging were performed. Retinal vascular status was evaluated by analyzing vascular density (VD) of superficial vascular plexus (superficial wVD) and deep vascular plexuses (deep wVD) in a 6 mm × 6 mm area and in foveal (superficial fVD and deep fVD) and parafoveal sectors (superficial pVD and deep pVD). In addition, foveal thickness (FT) and foveal avascular zone (FAZ) and clinical variables, including best corrected visual acuity (BCVA), intraocular pressure and refractive condition, were collected.

**Results:**

BCVA, intraocular pressure and refractive condition were comparable between cases and controls. OCTA imaging showed that superficial wVD and superficial pVD were lower in the psoriasis group in comparison with controls (*p* = 0.009 and *p* = 0.01, respectively). Similarly, deep wVD and pVD were lower in the psoriasis group in comparison with control subjects (*p* = 0.03 and *p* = 0.01, respectively). In a sub-analysis of 47 patients affected by psoriasis without psoriatic arthritis, lower values of wVD and pVD in both superficial and deep capillary plexuses were registered.

**Conclusion:**

OCTA is a useful tool which provides data on vascular status of the retina in psoriasis with no ocular involvement. VD data may suggest that vascular changes may occur earlier than clinical onset of posterior inflammation.

## Introduction

Psoriasis is a chronic inflammatory disease with a strong genetic background and autoimmune pathogenic traits. The pathogenesis of the disease has been associated to a dysregulation of TH1 and TH17 functions and balance that contributes to producing a systemic pro-inflammatory environment (Boehncke and Schön, 2015).

Ocular involvement is a common finding in psoriasis, it has been reported in up to 58% of patients (Kilic et al., 2013).

The most frequent manifestations are chronic non-specific conjunctivitis, blepharitis, dry eye, cataract, and anterior uveitis (Rehal et al., 2011). Psoriasis related uveitis has been associated with distinguishing clinical features. It is usually anterior, bilateral, and it often shows a relapsing course. Uveitis in patients affected by psoriasis is characterized by a high incidence of posterior involvement (Durrani and Foster, 2005). It includes inflammation of the choroid, retina, vitreous, and is often associated to visual impairment (Queiro et al., 2002; Fraga et al., 2012; Tanaka et al., 2015; Chi et al., 2017; Salek et al., 2018).

In several systemic inflammatory diseases with no clinical signs of ocular involvement, a vascular impairment of the retina has been shown (Dai et al., 2020; Pichi et al., 2020). Vascular assessment of the retina has been significantly improved by optical coherence tomography angiography (OCTA) (Spaide et al., 2018). OCTA is a novel non-invasive imaging technique which detects all vascular layers of the retina rapidly and with high resolution. OCTA is capable to identify early retinal vascular impairment in systemic disease, even in absence of clinical vitreoretinal and choroidal damage (Carnevali et al., 2017; Thompson et al., 2019).

To our knowledge this diagnostic approach has not been used on patients with psoriasis. The aim of the study is to assess morphological and vascular macular features in patients with psoriasis and controls using OCTA.

## Materials and Methods

In this prospective cross-sectional multicenter study, patients affected by psoriasis were recruited in two Departments of Dermatology (Dermatology Clinic of the University of Catania, Catania, Italy and Dermatology Clinic of the Federico II University, Naples, Italy) from July 2017 to July 2019. The protocol was approved by the Institutional Review Board of the participating centers and the procedures adhered to the tenets of the Declaration of Helsinki. A group of healthy subjects matched by age and sex, without any history of ocular and systemic diseases, were enrolled as controls.

Exclusion criteria were active or previous ocular inflammation, glaucoma, anamnesis of ocular trauma or surgery, high myopia ≥6.0 diopters, hyperopia ≥3.0 diopters, amblyopia, opacity of dioptric media, retinal, and optic disk clinical abnormalities, uncontrolled hypertension, diabetes and any ocular or systemic condition which could bias OCTA measurements. In patients with psoriasis the disease duration, the disease severity evaluated by Psoriasis area severity index (PASI score), and treatment with biologic therapy were evaluated. In addition, patient’s comorbidities were recorded. Patients and controls underwent an ophthalmic examination at the Ophthalmological Department of the University of Catania (Catania, Italy) or at the Ophthalmological Department of the Federico II University (Naples, Italy) including best corrected visual acuity (BCVA) on EDTRS charts, applanation tonometry, slit-lamp biomicroscopic examination with fundus dilation and OCTA. Only one eye per patient and control was enrolled in the study. OCTA examination was performed by the same expert ophthalmologist (A. L. Catania; G. C. Naples).

Optical Coherence Tomography Angiography of the macula was carried out employing AngioVue XR Avanti (Optovue Inc, Fremont, California, United States) after pupil dilation with 1% Tropicamide. The technology of OCTA has been previously described in detail (Querques et al., 2016; Moghimi et al., 2018; Bonfiglio et al., 2019; Rascunà et al., 2020). The rate of A-scan was 70,000 per second and the bandwidth was 50 nm. A light source centered on 840 nm was used. The acquired OCTA images of the macula (6 mm × 6 mm) were centered on the foveola. Each volume contained 400 × 400 A-scans with two consecutive B-scans captured in each fixed position. The dynamic motion of the red blood cells was captured using the split-spectrum amplitude-decorrelation angiography (SSADA) method. AngioVue software automatically segments the area into four layers, including superficial capillary plexus layer (SCP), deep capillary plexus layer (DCP), outer retinal layer and choriocapillaries (Spaide, 2015). OCTA automatic segmentation was validated by the operators (A. L. Catania, G. C. Naples). Vascular density (VD) was defined as the percentage of the area occupied by vessels. VD in the SCP and DCP were automatically calculated by the Optovue software with a density function. Foveal avascular zone (FAZ) area (mm^2^) was analyzed by the software with a non-flow function. The machine automatically provided data on foveal thickness (FT) concurrently to VD data. Macular whole image VD in the SCP and in the DCP (superficial wVD and deep wVD, respectively) measurements was calculated on the entire field of 6 mm x 6 mm scans centered on the fovea. In addition, the analyses of superficial and deep VD were focused on sub-sectors of the whole image including foveal (superficial fVD and deep fVD, respectively) and parafoveal (superficial pVD and deep pVD, respectively) area.

The images of poor quality with significant artifact and inappropriate segmentation at the level of the SCP and DCP were excluded from the analysis. A qualitative analysis at the level of the SCP and DCP was carried out including the following biomarkers: retinal blood flow loss, capillary remodeling and the evidence of retinal exudation.

All tested values included in the analysis were categorized as demographic data, OCTA data, ophthalmic characteristics and clinical data. Demographic data included sex and age. OCTA data included FAZ area, FT, superficial wVD, superficial pVD, superficial fVD, deep wVD, deep PVD, abd deep fVD. Ophthalmic characteristics included refractive error, intraocular pressure (IOP) and BCVA. Clinical data included the presence of arthritis in psoriasis patients.

The primary outcome measure of the study was to provide an assessment of the vascular status of the macula in patients with psoriasis in comparison to controls using OCTA. Secondary outcome measure was to evaluate the vascular status in a subset of psoriasis patients without arthritis.

The continuous variables were expressed as means and standard deviation (SD).

The values of different parameters detected in psoriasis and controls were compared using the unpaired *t*-test.

Values of P lower than 0.05 were considered statistically significant. Statistical analysis was conducted using the SPSS 21 soft package (SPSS, Inc., Chicago, IL, United States).

## Results

Overall, 55 eyes of psoriasis patients (32 male and 23 female) and 55 eyes of controls (32 male and 23 female) were included in the study. Complete demographic and clinical features are listed in Table 1. The mean ± SDs age of subjects was 52.4 ± 11.7 years in psoriasis group and 49.7 ± 7.9 years in control group. Eleven patients were affected by Hypercholesterolemia, eight patients had arterial hypertension controlled by medical therapy, three patients were obese (class 1). In psoriasis Group the mean duration of the disease was 6 ± 3 years, mean PASI score was 9.1 ± 5.4; 19 patients were treated by biologic therapy and 36 patients were treated by topical therapy. Between the two groups, no significant difference in BCVA, refraction and IOP values were found.

**TABLE 1:** Demographic and clinical characteristic of patients affected by psoriasis and control subjects.

	Psoriasis (*n* = 55)	Controls (*n* = 55)	Level of statistical significance (*p*)
Age (years), mean l’ SD	49.7 ± 7.9	52.4 ± 11.7	0.221
Gender (male:female)	32:23	32:23	−
BCVA (logMAR)	0.01 ± 0.04	0.02 ± 0.04	0.547
Refractive errors, (D), mean l’ SD	−0.4 ± 0.63	−0.2 ± 0.48	0.145
IOP (mmhg), mean l’ SD	14.4 ± 2.6	14.6 ± 2.5	0.660

*SD, standard deviation; BCVA, best corrected visual acuity; D, diopters; IOP, intraocular pressure.*

### Foveal Thickness, FAZ Area and Qualitative OCTA Analysis

As shown in Table 2, mean ± SD CFT were similar in the psoriasis group in comparison to the control group (258.7 ± 26.2 and 254.1 ± 19.4 micron, respectively; *p* = 0.351; *t*-test). Accordingly, no statistically significant difference in FAZ area between groups was shown (*p* = 0.775, *t*-test). None of the qualitative parameters included in the analysis (retinal blood flow loss, capillary remodeling and the evidence of retinal exudation) were detected either in the psoriasis or in control groups.

**TABLE 2:** Quantitative comparison of OCTA measurements between psoriasis group and controls.

	Psoriasis (*n* = 55)	Controls (*n* = 55)	Level of statistical significance (*p*)
FT (micron), mean l’ SD	258.7 ± 26.2	254.1 ± 19.4	0.351
FAZ, (mm^2^), mean l’ SD	0.263 ± 0.11	0.270 ± 0.11	0.775
Superficial wVD (%), mean l’ SD	46.9 ± 3.7	48.8 ± 3.1	0.009
Superficial fVD (%), mean l’ SD	20.5 ± 6.6	20.3 ± 7.2	0.884
Superficial pVD (%), mean l’ SD	48.2 ± 4.8	50.5 ± 3.7	0.014
Deep wVD (%), mean l’ SD	47.5 ± 6.2	50.1 ± 5.2	0.034
Deep fVD (%), mean l’ SD	36.6 ± 7.9	37 ± 7.8	0.792
Deep pVD (%), mean l’ SD	51.6 ± 4.9	54.1 ± 3.9	0.012

*FT, foveal thickness; SD, standard deviation; FAZ, foveal avascular zone; wVD, whole image vascular density; fVD, foveal vascular density; pVD, parafoveal vascular density.*

### OCTA Measurements of the Superficial Capillary Plexus

**FIGURE 1:**
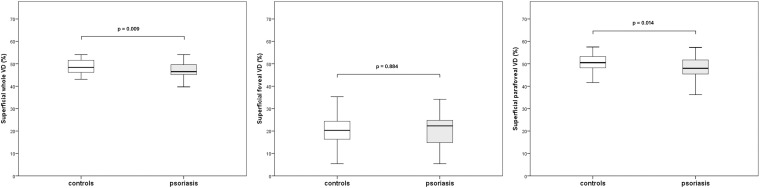
Boxplots illustrating vascular density (VD) of the superficial capillary plexus in the psoriasis group and controls.

The mean ± SD values of superficial wVD were lower in the psoriasis group in comparison with controls (46.9 ± 3.7 vs 48.8 ± 3.1, *p* = 0.009, *t*-test). Similar outcomes were detected in superficial pVD (48.2 ± 4.8 vs 50.5 ± 3.7, *p* = 0.01, *t*-test) (Figure 1). No statistically significant difference in the superficial fVD was observed. Complete data on vessel density are listed in Table 2.

### OCTA Measurements of the Deep Capillary Plexus

**FIGURE 2:**
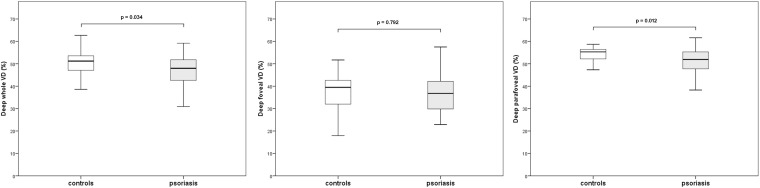
Boxplots illustrating vascular density (VD) of the deep capillary plexus in the psoriasis group and controls.

**FIGURE 3:**
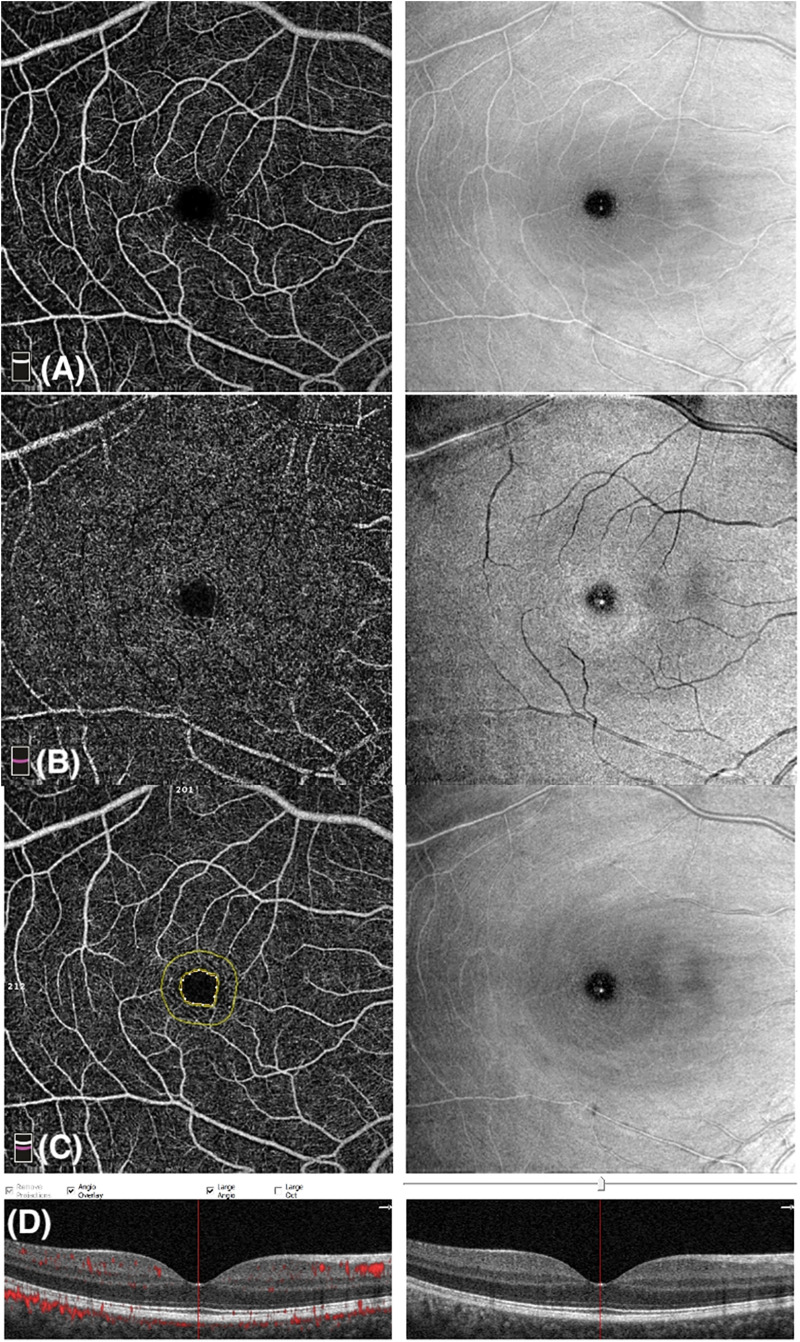
Optical coherence tomography angiography (OCTA) image of a control subject with a scan size of 6 × 6-mm. **(A)** Enface angiogram segmented at the level of the superficial capillary plexus (SCP). **(B)** Enface angiogram segmented at the level of the deep capillary plexus (DCP). **(C)** Foveal Avascular Zone (FAZ): area encircled by the inner yellow line; the outer yellow line is 300-μm far from the FAZ. **(D)** B-scan retinal image shows blood flow at the level of SCP, DCP, and choriocapillaris.

**FIGURE 4:**
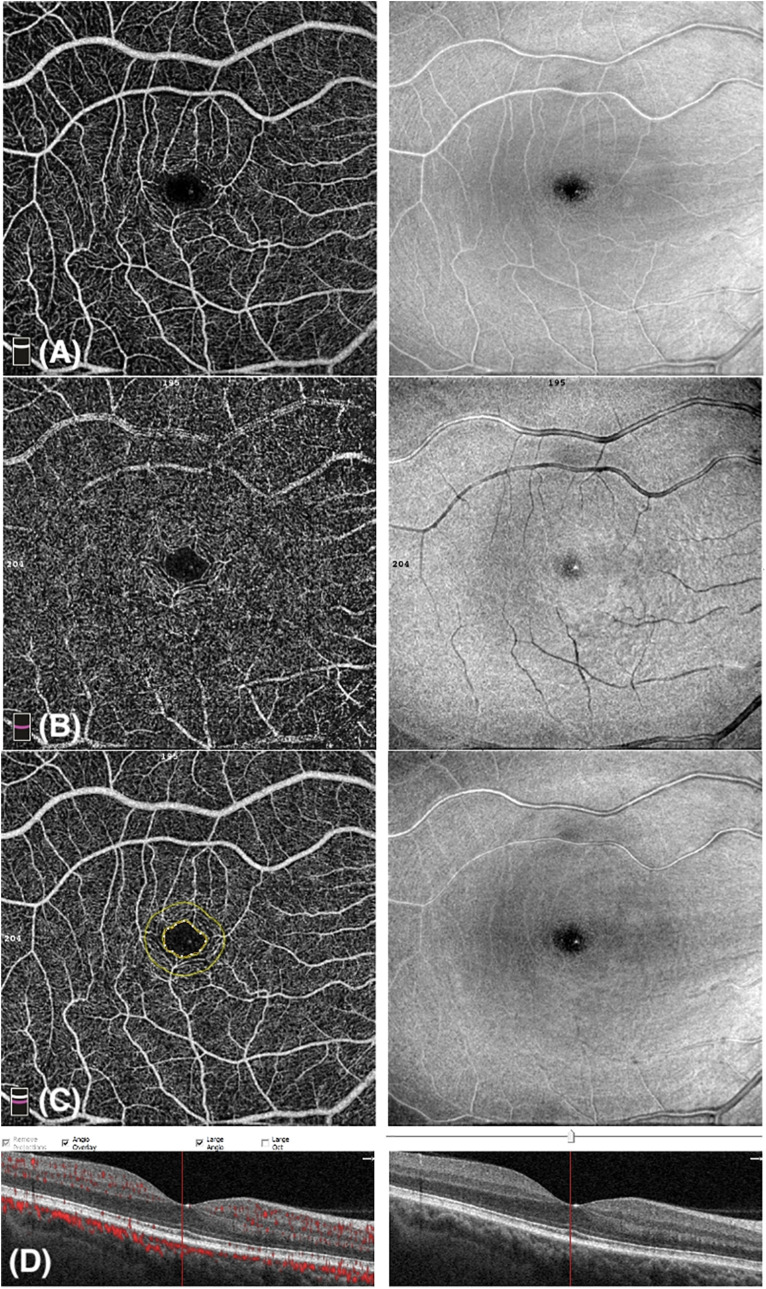
Optical coherence tomography angiography (OCTA) image of a patient affected by Psoriasis with a scan size of 6 × 6-mm. **(A)** Enface angiogram segmented at the level of the superficial capillary plexus (SCP). **(B)** Enface angiogram segmented at the level of the deep capillary plexus (DCP). **(C)** Foveal Avascular Zone (FAZ): area encircled by the inner yellow line; the outer yellow line is 300-μm far from the FAZ. **(D)** B-scan retinal image shows blood flow at the level of SCP, DCP, and choriocapillaris.

Whole image vessel density of the deep capillary plexus in the psoriasis group was significantly lower in comparison with control subjects (47.5 ± 6.2 vs 50.1 ± 5.2, respectively, *p* = 0.03, *t*-test). Lower values of deep pVD in the psoriasis patients in comparison with the control group (51.6 ± 4.9 vs 54.1 ± 3.9, respectively, *p* = 0.01, *t*-test) were registered. No statistically significant difference in the deep fVD was observed. Figure 2 graphically shows VD measurements of the DCP. Representative images of OCTA examination in control subjects and psoriasis patients are shown in Figures 3, 4, repectively.

### OCTA Measurements in Patients Without Psoriatic Arthritis

Eight psoriasis group patients were affected by arthritis. In the sub-analysis of 47 eyes belonging to 47 patients affected by psoriasis without psoriatic arthritis, the outcomes registered were similar to those from the primary outcome measure if compared to the control group. In detail, lower values of wVD and pVD in both superficial and deep capillary plexuses were registered. Complete data are listed in Table 3.

**TABLE 3:** Quantitative comparison of OCTA measurements between psoriasis without arthritis group and controls.

	Psoriasis without arthritis (*n* = 47)	Controls (*n* = 55)	Level of statistical significance (*p*)
FT (micron), mean l’ SD	255 ± 26	254.1 ± 19.4	0.861
FAZ, (mm^2^), mean l’ SD	0.260 ± 0.11	0.270 ± 0.11	0.801
Superficial wVD (%), mean l’ SD	47.4 ± 3.2	48.8 ± 3.1	0.045
Superficial fVD (%), mean l’ SD	21 ± 6.8	20.3 ± 7.2	0.601
Superficial pVD (%), mean l’ SD	48.7 ± 4.3	50.5 ± 3.7	0.048
Deep wVD (%), mean l’ SD	47.7 ± 5.7	50.1 ± 5.2	0.047
Deep fVD (%), mean l’ SD	36.9 ± 8	37 ± 7.8	0.919
Deep pVD (%), mean l’ SD	52.1 ± 4.1	54.1 ± 3.9	0.025

*FT, foveal thickness; SD, standard deviation; FAZ, foveal avascular zone; wVD, whole image vascular density; fVD, foveal vascular density; pVD, parafoveal vascular density.*

## Discussion

In this study, where we employed the OCTA on patients with psoriasis without any history of uveitis, we observed a reduction of VD in comparison to controls. Psoriasis is a systemic inflammatory disease associated with a dysregulation of TH cell function. Previous authors have described psoriasis as a systemic disease with a propensity for skin, joint and eye involvement (Durrani and Foster, 2005). Ocular findings in patients with posterior psoriatic uveitis included heavy vitreal debris, retinal exudation, retinal vasculitis and cystoid macular edema (Knox, 1979; Rehal et al., 2011). Choroidal involvement in psoriasis has been widely investigated using optical coherence tomography, even in patients affected by psoriasis without any history of uveitis. Ersan et al. (2017) in a recent study on psoriasis patients with no signs of posterior uveitis, reported an increased subfoveal choroidal thickness in patients with severe psoriasis, while no difference was found by analyzing the macular thickness and macular ganglion inner cell plexiform layer thickness. Accordingly, Türkcü et al. (2016) described similar outcomes by detecting an increased choroidal thickness.

At the best of our knowledge, no data are available on the assessment of retinal vasculature by OCTA in patients with psoriasis.

In the present study, lower values of VD in both superficial and deep capillary plexuses were detected in psoriasis patients, when compared to controls. More in detail, VD in those patients was significantly reduced on the whole area of the image and on the parafoveal sector of the superficial vascular plexus. Similarly, deep wVD and deep pVD were significantly lower in psoriasis group patients in comparison to controls.

Vascular status of the retina has been investigated by OCTA in other inflammatory systemic pathologies with no evidence of ocular involvement. Pichi et al. (2020) reported lower values of VD in the superficial capillary plexuses in systemic lupus erythematosus patients. Blood flow in retinal vascular plexuses is dysregulated in the course of inflammation and OCTA has been identified as a sensitive tool for evaluating the vessel modification related to inflammation. Retinal vascular and perivascular alterations due to uveitis were demonstrated by OCTA (Invernizzi et al., 2019) reporting lower values of VD in both superficial and deep capillary plexuses. In our study we excluded patients with any evidence of uveitis. It should be kept in mind that in the pathogenesis of psoriasis several cytokines are involved and cause dysregulation of endothelial cell function (Di Cesare et al., 2009; Deng et al., 2016). The inflammation of posterior segment in psoriasis is complex and not fully understood. We speculate that our results are related to the subclinical inflammation involving retinal microvascular blood flow. Recently, Fragiotta et al. (2020) demonstrated that patients affected by psoriasis without uveitis, in comparison to control subjects, are characterized by reduced arterovenus ratio in the superficial retinal vessels and reduced thickness of the choriocapillaris. These microvascular alterations, detected by OCT, were associated with the upregulation of a subpopulation of T cells. On the other hand, the authors did not find changes of pro-inflammatory cytokine levels in the aqueous humor. However, inflammatory cytokine levels may be downregulated by immunomodulatory therapy, which is employed in a significant number of psoriasis patients.

Several studies have been shown the association between uveitis and psoriatic arthritis (Lambert and Wright, 1976; Salek et al., 2018). The association between uveitis and psoriasis in patients without psoriatic arthritis has been debated because limited data were available. Egeberg et al. (2015) have been clarified the association between psoriasis and uveitis. The authors have shown an increased risk for uveitis both in patients with psoriatic arthritis and psoriasis patients without arthritis. Accordingly, in our study, even in the sub-analysis of psoriasis patients with no evidence of psoriatic arthritis lower data of VD in both superficial and deep capillary plexuses were observed in comparison with controls. Therefore, the present study may provide additional data to the concept that inflammatory changes of eye blood vessels are associated to psoriasis, even in absence of arthritis.

Optical Coherence Tomography Angiography, in addition to its diagnostic and clinical management role, may represent an important tool to contribute to the pathophysiological knowledge of retinal changes in systemic disease.

The present study has several limitations including the cross-sectional nature, the absence of a longitudinal follow-up, the unavailability of biochemical data, the small sample of the arthritis patient subgroup and the lack of choroidal assessment.

In conclusion OCTA is a useful tool for the assessment of retinal vascular status in psoriasis. Vascular changes were found in psoriasis patients without clinical signs of ocular inflammation. Whether these changes could be early findings that might anticipate the development of ocular inflammation needs to be investigated by further studies.

## Data Availability Statement

The raw data supporting the conclusions of this article will be made available by the authors, without undue reservation.

## Ethics Statement

The studies involving human participants were reviewed and approved by the Institutional Review Board Catania. The patients/participants provided their written informed consent to participate in this study.

## Author Contributions

NC, MR, and AL: conceptualization. TA, GM, GF, and MF: methodology. GP and AR: formal analysis. PA, FP, RD, and FF: data curation. NC: writing—original draft preparation. NC, MF, VB, and AL: writing—review and editing. FL, MM, GC, and MR: supervision. All authors contributed to the article and approved the submitted version.

## Conflict of Interest

The authors declare that the research was conducted in the absence of any commercial or financial relationships that could be construed as a potential conflict of interest.
